# Interactive, open source, travel time scenario modelling: tools to facilitate participation in health service access analysis

**DOI:** 10.1186/s12942-017-0086-8

**Published:** 2017-04-18

**Authors:** Rohan Fisher, Jonatan Lassa

**Affiliations:** Charles Darwin University, Ellengowan Dr, Casuarina, NT 0810 Australia

## Abstract

**Background:**

Modelling travel time to services has become a common public health tool for planning service provision but the usefulness of these analyses is constrained by the availability of accurate input data and limitations inherent in the assumptions and parameterisation. This is particularly an issue in the developing world where access to basic data is limited and travel is often complex and multi-modal. Improving the accuracy and relevance in this context requires greater accessibility to, and flexibility in, travel time modelling tools to facilitate the incorporation of local knowledge and the rapid exploration of multiple travel scenarios. The aim of this work was to develop simple open source, adaptable, interactive travel time modelling tools to allow greater access to and participation in service access analysis.

**Results:**

Described are three interconnected applications designed to reduce some of the barriers to the more wide-spread use of GIS analysis of service access and allow for complex spatial and temporal variations in service availability. These applications are an open source GIS tool-kit and two geo-simulation models. The development of these tools was guided by health service issues from a developing world context but they present a general approach to enabling greater access to and flexibility in health access modelling. The tools demonstrate a method that substantially simplifies the process for conducting travel time assessments and demonstrate a dynamic, interactive approach in an open source GIS format. In addition this paper provides examples from empirical experience where these tools have informed better policy and planning.

**Conclusion:**

Travel and health service access is complex and cannot be reduced to a few static modeled outputs. The approaches described in this paper use a unique set of tools to explore this complexity, promote discussion and build understanding with the goal of producing better planning outcomes. The accessible, flexible, interactive and responsive nature of the applications described has the potential to allow complex environmental social and political considerations to be incorporated and visualised. Through supporting evidence-based planning the innovative modelling practices described have the potential to help local health and emergency response planning in the developing world.

## Background

Proximity to health services is a key factor determining outcomes for a range of health issues [1–4] including ongoing treatment for chronic conditions [4], preventative services [5] and emergency response [6]. Over the last decade therefore, as spatial data and geographic information systems have become increasingly available, considerable attention has been given to understanding the geographic dimensions of access [7, 8]. This work has largely focused on identifying populations remote from health services through the spatial modelling of travel time and has become a common planning tool supporting transport and service infrastructure planning [7].

The usefulness of such spatial decision support systems (SDSS) are however constrained by the availability of accurate input data and limitations inherent in the assumptions, parameterisation and methods used [2, 9–11]. Furthermore these analysis have been criticised for being developed in a top–down way that does not allow for the incorporation of qualitative data or individual experiences [10, 12]. These limitations have led to the value and utility of these forms of analysis being questioned when considered within a broader framework of health service provision that has to factor in a complex array of dynamic environmental, social and economic determinants [9, 12, 13]. Indeed it has been argued that current *empirical* health studies modelling accessibility tend to over simplify access in complex health care landscapes, leading to misinformed policy interventions [10]. Various authors have called for studies that provide a more nuanced understanding of the relationship between specific populations and their unique geographical contexts [10, 14, 15]. Neutens [10] specifically argues for individual-based and temporally integrated analysis and more sophisticated geo-computational tools that address social disparities in accessibility of health care. Along with new spatial analytical tools, it has also been suggested that new geo-visualisation methods are needed to enable a more sophisticated evaluation of complex multidimensional travel with greater flexibility to facilitate the incorporation of local knowledge [8, 12, 15]. Currently however, these modelling tools are primarily available only in proprietary GIS or SDSS packages requiring considerable expertise to operate, and produce static outputs failing to capture the complexity of individual travel patterns [10, 16]. Local health planners in developing countries often need much simpler approaches to SDSS. The lack of accessibility to, and flexibility of, these tools is particularly an issue in the developing world where human and financial resources are limited, as is access to basic spatial data [17], also travel is often complex and multi-modal [13].

The aim of the work presented in this paper was to develop simple, open source, adaptable and interactive service access modelling tools that improve access to health services through facilitating more sophisticated multi-temporal modelling, the incorporation of local knowledge and supporting participatory planning. While the development of the tools described in this paper was guided by health service issues from a specific developing world context, the approach described has utility, more generally for enabling greater access to, and more nuanced, health service planning and effective emergency medicine. The following section explores some of the underlying assumptions and limitations in standard forms of travel time modelling and their relevance in a health service delivery context. Modelling tools designed to address some of the identified limitations are then described.

### Assumptions and limitations in health service access analysis

Most travel analysis does not take into account the socio-economic or health conditions of individual cases, differential access to transport infrastructure (e.g. car ownership), or temporal variability in travel and access as well as different scenarios for disruptions and delays resulting from natural hazards. This is because the spatial analysis methods make rigid assumptions that are unlikely to hold in real life. Examples include the assumptions that all people walk or drive at the same rate, that motorised transport is immediately available when transport networks are reached, and that travel and service availability conditions remain constant irrespective of time of day or weather conditions [2, 10, 13]. Furthermore, in many of these analyses, the underlying assumptions and parameter settings are developed in a top–down way, not clearly described or understood by planners, and hide significant variability [2]. As a consequence of the inability of these analyses to capture the complexity of real travel it is suggested that only average scenarios can usefully be modelled [2]. Conversely it is argued that such aggregated or averaged analysis, by ignoring important sources of social inequality, and diverse often gendered health needs, can result in planning that does not take into account the most at-risk groups [9, 10, 18]. The methods described in this paper were initially developed to assess access to emergency obstetric care (EMOC) in rural Indonesia where averaged measures of travel fail to capture the complex, interacting variables involved in accessing care [13]. In this example reducing high maternal mortality rates requires understanding extreme, although still common, individual cases of travel of usually poor young women.

In summary two issues commonly poorly addressed in most health service access analysis are (1) the inherently multi-modal nature of travel for people living away from regular transport networks, with limited access to private transport and/or health conditions affecting mobility and (2) the temporal variability in transport and service access. Two additional factors limiting the utility of these forms of analysis, more often associated with the developing country contexts, are a lack of basic health and transport infrastructure data and the requirement for significant human and financial resources to conduct travel time analysis [17]. Many studies modelling travel time to health services cite one or more of these limitations affecting their analysis [1, 4, 19–21].

The inclusion of temporal variation in the availability of services and travelling conditions has been previously described as a key factor that is often over looked when considering access to services [9]. Similarly seasonal and weather conditions have been shown to be a critical factor, particularly in relation to emergency access and response [13]. Service access models also rarely factor in temporal variation in staff, equipment or medicine availability [9]. Combining temporal variation with multiple modes of travel produces a complex multidimensional array of access scenarios. For example even a simple scenario range involving three patient conditions (walking, walking aided, requiring stretcher), two time-of-day factors (Night, Day), three weather influences (Dry, Rain, Heavy Rain), and two availability of service factors (doctor in or away on call) results in 36 possible model outputs. Currently however most travel time analysis account for no more than a few possible travel time scenarios resulting in a simplified and potentially misleading vision of the multi-dimensional nature of service access.

Service access studies are also constrained by the method of analysis used. Travel modelling generally employs either network or raster based spatially analytical techniques [2]. Network analysis uses optimised transport infrastructure networks to calculate travel and allows for the incorporation of a complex array of rules governing transport movement. Whilst more accurate than Euclidian, distance this method does not take into account off network travel which is an important factor, particularly in remote rural regions of the developing world where there may be little transport infrastructure and walking is required [1, 2, 22, 23]. Off network or across network walking can also be a substantial component of travel in industrial urban settings [11]. In contrast raster based analysis, by calculating potential travel speed for all cells in a gridded study area, allows both network and off-network modes of travel to be considered and is thus the preferred method for analysis in remote rural regions of developing countries[22–25].

Finally the ability to model travel time is often further limited, particularly in developing country contexts, by a paucity of basic up-to-date transport and health infrastructure data, detailed population distribution information and knowledge of local travel habits. This information, however, often exists in the form of local knowledge derived through lived experience. It has been suggested that facilitating participatory engagement in this modelling would allow the incorporation of local knowledge critical for bridging gaps in available information [13]. In addition, building participatory analysis tools would more effectively capture a useful range of travel time scenarios and allow greater transparency around model assumptions [13, 17].

### Scenario modelling tools

The tools developed through this work are significantly different from currently available travel time tools that are largely only available in expensive proprietary software, are complex to set-up and do not support dynamic interactivity. Three interconnected applications were developed designed to overcome some the described limitations and bridge the gap between travel time analysis and local participatory planning. These are; an open source tool-kit for raster based travel time analysis and two interactive geo-simulations allowing rapid, interactive service access scenario modelling. Although the tools described emphasise travel time variables derived from work conducted previously in monsoonal Eastern Indonesia, many of the variables used and processes described are universal to most health access contexts. Of greater significance than the health service example in this study is the open and adaptable nature of these applications whereby alternative factors affecting service access can be easily emphasised or incorporated as appropriate to a specific context.

Through these applications this paper aims to explore novel approaches to enable the decentralised application of travel time modelling for evidence-based infrastructure planning through the creation of interactive modelling tools.

#### GIS toolkit

Most of the travel time modelling tools currently available are in expensive, complex, proprietary GIS software limiting their decentralised application. These tools are also limited in their ability to support rapid multiple scenario creation. However, the increasing availability of high quality free open source (FOS) geospatial software is opening new opportunities for supporting local participatory planning processes and collaborative decision making [26]. Furthermore the increasing availability of free satellite imagery, elevation data and raster based spatial analysis software is providing new opportunities for local stakeholders to combine the quantitative analysis of ‘hard’ data with qualitative local knowledge [27]. Described in this paper is a tool kit developed for SAGA-GIS software that automates and simplifies some of the processes required for conducting raster based travel time analysis. This tool kit also allows a degree of interactivity and adaptability supporting the rapid exploration of multiple travel time scenarios through altering continuous variables, such as travel speed as well as adding discrete factors such as barriers, new roads or service provision. The GIS based travel time tool, similar to the approach developed by the WHO through their Access Mod plugin [28], is designed to facilitate raster-based travel time analysis through automating and simplifying the processing steps involved.

#### Geo-simulation applications

Building on the SAGA based application, additional development was conducted in the free open source NetLogo Agent-based modelling (ABM) platform to produce a spatially explicit cellular automata model. This form of modelling, also refereed to geosimulation [29–31], allows for the simulation of complex systems integrating spatial data to explore problems of geography, the environment, planning and social systems. Key advantages to an ABM or geosimulation approach to modelling are that they support interactive, iterative experimentation with model parameter settings through a user friendly interface, they enable the simple automation of model runs based on a range of parameter settings to rapidly produce a multidimensional array of possible scenario outcomes and they allow the integration of the temporal dimension into normally static GIS outputs. For health planning ABM or geo-simulation approaches have been used for a wide variety of applications including improving health care delivery systems [32, 33], supporting investigations into neighborhood walk ability [16] and understanding disease transmission [34].

For the purposes of this paper, the two applications produced in NetLogo are referred to as geo-simulations. The first incorporates the travel time calculation into the geo-simulation and allows the rapid exploration of multiple travel time scenarios through a user friendly interface. This model facilitates dynamic, participatory travel time modelling for exploring impacts on access to care through enabling changes to continuous variables (such as rainfall or travel speed), and the addition of discrete factors (such as road breaks or new services). The second geo-simulations adds transport network agents (cars), demonstrating the integration of raster and network forms of travel, and allows the interactive placement of patient agents simulating individual travel cases. In both cases the geo-simulations format removes any need for GIS or modelling skills through creating a simple and accessible format for interactive scenario modelling.

### Methods

**Fig. 1:**
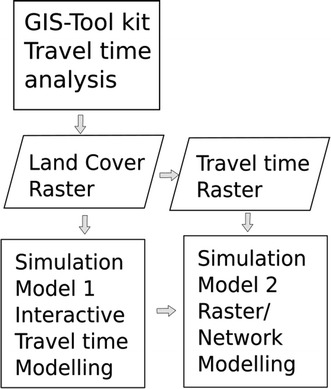
A conceptual diagram showing the links between the GIS tool-kit and the geo-simulation models described

The open source GIS Toolkit for service access analysis outputs two spatial datasets. The first is a Land cover grid which combines landscape features and transport infrastructure, the second uses this layer to model travel time to defined health care points. These data sets are then incorporated into the geo-simulation models. The land cover raster is used as a base layer for travel time modelling in the first geo-simulation whilst the second geo-simulation incorporates a travel time raster produced by either the GIS-toolkit or the first geo-simulation model as shown in Fig. 1.

### GIS tool kit

SAGA GIS was used for this application as it provides a comprehensive suite of raster analysis tools, including satellite image classification, hydrological modelling, cost distance analysis and a sophisticated tool scripting function that allows the integration and automation of processes [35]. The SAGA Tool Chains capability uses a simple xml scripting code that allows the joining of multiple SAGA processes. The two tools were produced (1) for land cover grid creation and (2) travel time calculation, the latter requiring the output of the first as shown in Fig. 1. Separating the two tools allows for the testing of multiple scenarios with the Travel Time Grid creation tool without having to recalculate the base land cover grid each analysis iteration.

#### Land cover grid creation

The Land Cover grid creation tool combines vegetation, roads and elevation data to produce a raster grid of land cover, transport infrastructure and watercourses. All of these base layers were derived from free geospatial datasets. The land cover grid is used as the basis for the travel time calculation with each land cover type being allocated a potential travel speed value. In the example shown here the vegetation, road and elevation data were processed as follows.

Vegetation data was derived from Landsat 8 satellite imagery. An unsupervised classification was conducted and assigned to four broad classes; Forest, Scrub, Grassland, Bare Land, based on local knowledge and Google Earth imagery. This data set was produced at a 50 m grid cell size and became the base grid system to which other created data were resampled. The 90 m resolution NASA SRTM digital elevation dataset [36] was used to produce a watercourses grid within the tool chain. Roads were obtained from Open Street Map [37] as vector data and attributed into three classes based on infrastructure quality; national, provincial and local roads.

**Fig. 2:**
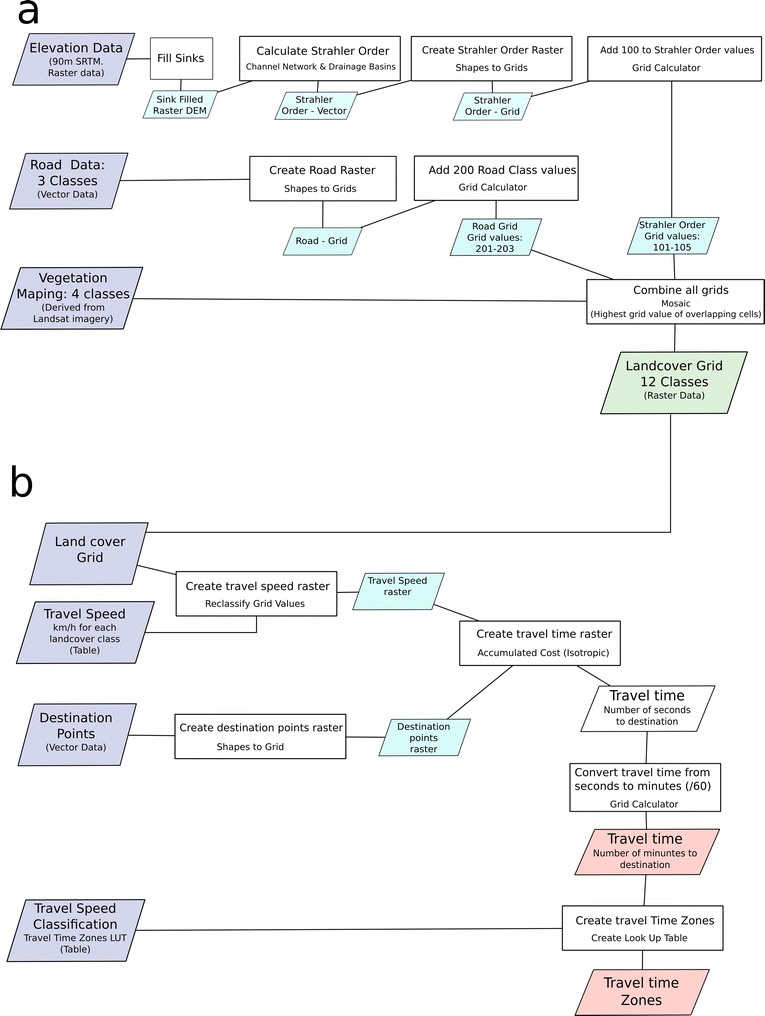
Flow chart of travel time processing within the SAGA chain tool application showing **a** the joining of vegetation, elevation and road data to produce a land cover grid and **b** the integration of this land cover layer with land cover travel speed look up tables and destination points to produce the final travel time grid

These three data sets (Vegetation, Roads and Elevation) were the base inputs into the Land cover tool chain which produced the final land cover described below and shown in Fig. 2a. The elevation data set was used to produce a raster grid of water courses by; (1) implementing a pre-processing (Fill Sinks) operation (2), from this producing stream network vector data attributed with Strahler order and(3) finally converting this to a raster grid. The Strahler order is a measure of flow accumulation within a landscape used to model barriers to travel based on different seasonal scenarios for travel time calculation. Five Strahler classes were produced in the analysis grid with the highest class unlikely to be passable at anytime of year. The road data was then converted to a raster grid mosaicked together with the Strahler order and vegetation data to produce one grid with twelve classes.

#### Travel time grid calculation

The Travel Time Grid creation tool produces two output grids; (1) travel time to destination in minutes and (2) this data reclassified as travel time or remoteness zones. The Travel Time Grid creation tool requires the input of the Land Cover grid, destination points as vector data, and two reclassification tables. The first reclassification table gives travel speed values for each land cover class calculated as the number of seconds to travel across one grid cell. Table 1 shows the twelve land cover grid classes with example travel speed values. The second table defines a look up table (LUT) reclassing the output grid into travel zones (Table 2). The destination data is provided as vector points.

**Table 1:** Reclassification table showing cover classes, travel speed and travel time

ID	Cover class	km/h	Travel time (s)
1	Forest	1	180
2	Grass	2	90
3	Bare, rocky	3	60
4	Scrub	0.75	240
101	Stream class 1	2	90
102	Stream class 2	2	90
103	Stream class 3	2	90
104	Stream class 4	2	90
105	Stream class 5	0	99,999
201	National road/Hi-way	50	3
202	Provincial road	25	7
203	Local road/track	10	18

**Table 2:** Travel time zone reclassification table

Class	Description (min)	Minimum	Maximum
1	0–15	0	15
2	15–30	15	30
3	30–60	30	60
4	60–90	60	90
5	90–120	90	120
6	120+	120	999,999

Using these data the travel time tool chain first creates a new grid with each cell attributed the travel cost in seconds as shown in Fig. 2b. Using an isotropic cost distance analysis, a grid of travel time in seconds to destination points is then produced. Finally this is converted to travel time in minutes and reclassified to travel zones using the second reclassification table.

#### Interactive modelling

Once the initial travel time grid is produced, alternative travel time scenarios can be rapidly modelled by: changing the travel speed table, altering the location and number of destination points and editing the Land Cover grid.

Changing the travel speed values for each land cover class in the travel speed reclassification table allows the rapid exploration of a range of potential travel time scenarios. For example you may wish to look at travel time after different intensity rain events by giving some or all of the watercourse classes (101–105) a very high value (>99,999) so they form a travel barrier due to flooding. Other examples, as mentioned in the introduction, may include altering speeds for walking off transport networks (assisted or carried) or by time of day travel.

Destination points can be quickly added, moved or deleted as a way of altering service availability scenarios. Disruptions and delays can be also added for example. This could be used to model a range of service option scenarios from the building of a new clinic to the simple provision of a piece of equipment and/or staff capacity to meet a particular health need.

Within SAGA it is also possible to directly edit the Land Cover grid using an interactive tool to change cell values. This enables drawing new land cover values directly onto a grid, thus changing the potential travel speed for an individual cell. This can be used, for example, to reduce travel speeds on sections of road known to be damaged or add new roads and tracks. Drawing road damage or adding roads known to exist but not in official data allows the direct input of local knowledge to improve the accuracy of the model. A video demonstration of the SAGA tool use and additional tutorial material can be accessed at https://rohanfisher.wordpress.com/travel-time-modelling-saga/.

### Geo-simulation models of travel time

NetLogo was chosen as the simulation modelling platform for this application as it provides a relatively simple coding format easily shared and learnt and provides a comprehensive set of GIS data integration tools [38]. Two approaches for integrating the SAGA derived GIS data into the ABM platform are described here. The first model uses the land cover output grid and conducts the travel time analysis with NetLogo. The second model described here uses the final travel time data produced from SAGA for the interactive visualisation of individual travel time cases.

#### Geo-simulation 1 (distance transform implementation)

This model explores the integration of cost distance spatial analysis techniques into a simulation platform to enable more interactive travel time modelling. It duplicates the interactive modelling features described in the SAGA tool in a more accessible form with an intuitive user interface. This model uses the Land cover grid produced by the Land cover chain tool and calculates travel using a cost distance transform coded into Netlogo.

To initialise the model, a travel speed grid is first calculated from a look up table that integrates inputs via the user interface (Fig. 4) with the land cover grid. An accumulated cost grid is also created with all cells initially allocated a default value of 99,999 and with destination points allocated a value of 0. Based on these grids a standard Chamfer metric algorithm is used to calculate the accumulated cost grid [39, 40]. The chamfer metric technique uses four scans of the travel speed grid with a 5 × 5 filter. The 5 × 5 distance transform mask calculates the fractional distance from the centre point and produces a substantially more accurate distance estimate than pure local (3 × 3) Euclidean filters.

**Fig. 3:**
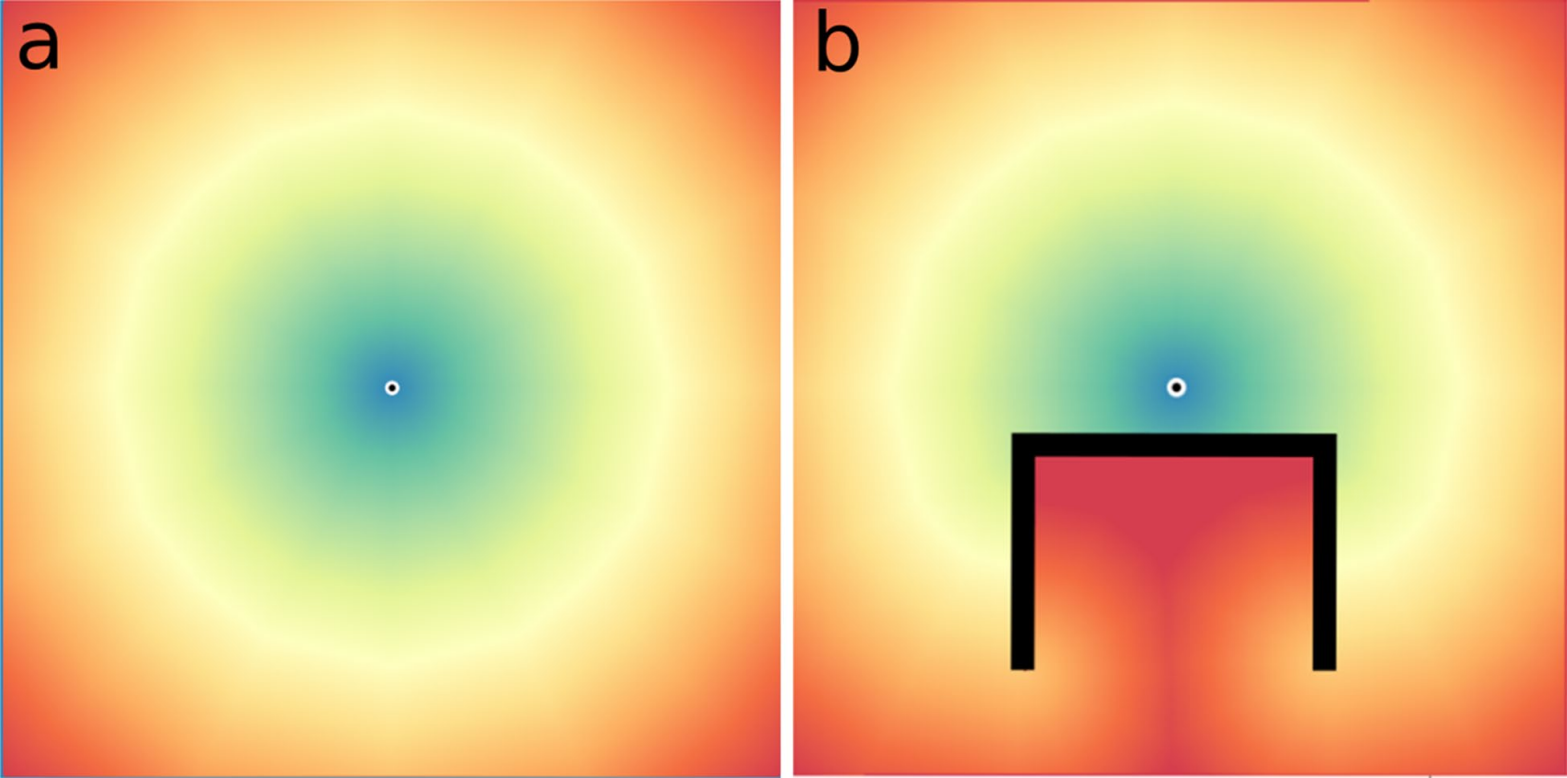
Output from the distance transform ABM show travel time to a centre point **a** unobstructed and with **b** an impassable barrier

The scans are conducted by a model agent moving over the cost grid first vertically from top-left to the bottom right corner then backwards bottom right to top left then repeated as a horizontal scan. Four scans where found to produce an accurate model output, additional scans where found to increase accuracy marginally at a substantial cost of additional processing time. At each point of the scan the travel time from the central cell to all surrounding cells is calculated using the travel speed values and multiplied by the fractional value, derived from Smith et al. [40]. The minimum calculated travel time of surrounding cells is then added to the current cell travel time value if it is smaller than the current value. A basic output from the Netlogo model is shown in Fig. 3a with a constant travel speed and one central destination point. Figure 3b shows the same output with an impassable barrier drawn onto the raster grid.

The example implementation allows the grid cell values to be changed by placing destination points, drawing barriers or roads, time of day to be set, walking mode and road speeds to be to be altered and different weather conditions to be explored.

**Fig. 4:**
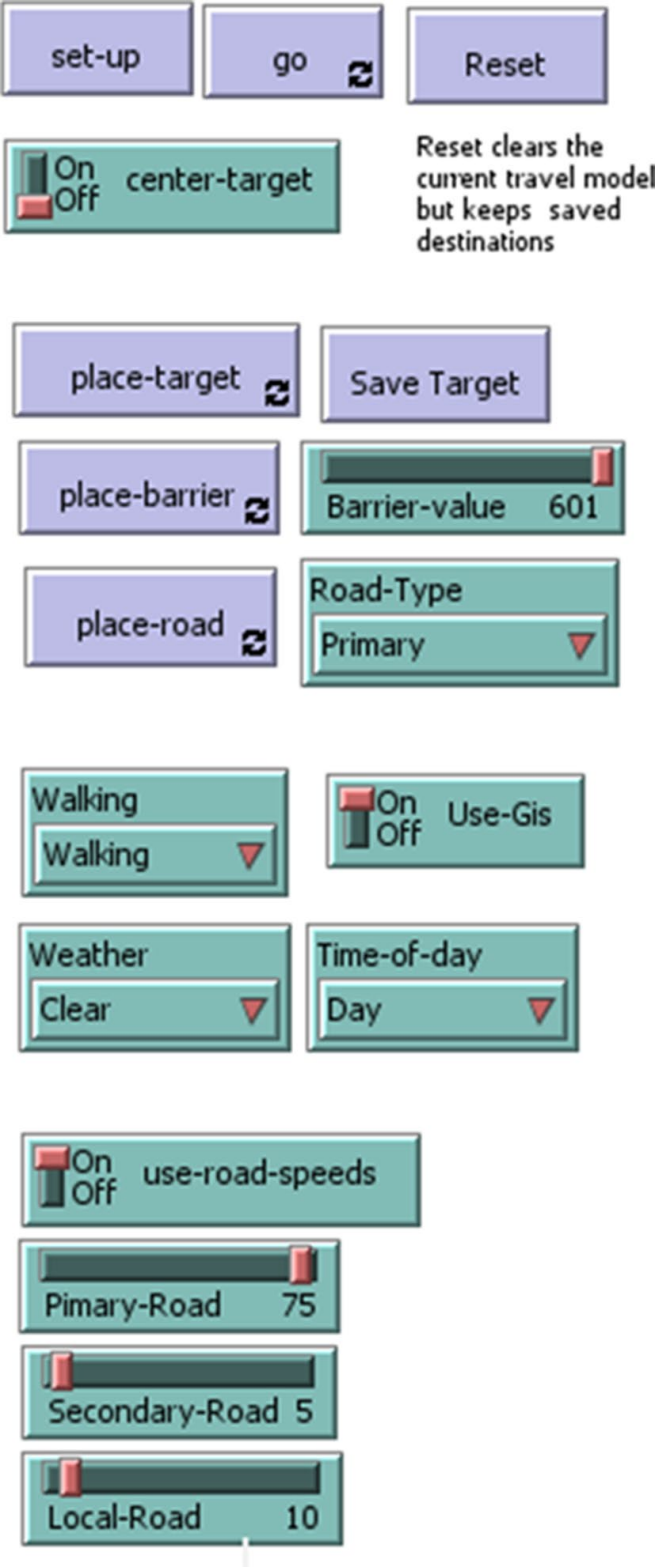
Travel parameter setting interface in the Netlogo distance analysis model

Once the model is initialised various model parameters can be modified through the interface shown in Fig. 4. Placing destination points (place target) sets the accumulated cost value to 0 of grid cells using the mouse interaction. Set points can be saved (save target) so the same destinations can be used for multiple runs of the model with different parameter settings. Barriers can be drawn with variable barrier intensity selectable by a slider that sets the new travel speed from 1 to 600 min for selected cells. This can be used, for example, to slow travel on roads that are damaged or to place completed breaks in roads. Roads can be drawn by selecting one of the three road classes in the drop down list. This changes the land cover grid to match the selected road class value. Road type speeds can also be altered individually using the ‘use road speeds’ option. This allows customising of the travel speeds overriding set values in the model. This can be useful to model and compare different types of vehicle transport, i.e. comparing bus travel to ambulance travel. Finally, if the time of day variable is set to night, all travel is slowed by twenty percent.

Altering the walking mode halves the walking speed if set to ‘assisted’ and cuts walking speed by a 4/5ths if set to ‘stretcher’. The weather can be altered to ‘rainy’ which makes the three highest Strahler order water courses impassable by setting the travel time value to 99,999, and ‘flooding’ sets all the water courses to impassable.

#### Geo-simulation model 2 (raster-network)

This model uses the travel time output grid from the SAGA tool chain whereby all the cells are attributed a distance to a destination point. This model has two primary purposes. The first is to allow the placement of individuals to ‘game’ particular travel scenarios. An individual representing a travel time case moves at each cycle of the model to the next smallest cell value, essentially ‘flowing’ from the highest starting value to the lowest sink destination value. The second aim of this model is to explore the integration of a transport network form of travel into a raster model environment.

This mode adds the variable and random nature of car traffic and the fact that transport is not always immediately available particularly in remote rural communities as it is assumed to be when using the raster surface alone. When the ‘Use Cars’ function of the model is activated, car agents are added traveling only on road classes always moving to a destination point. When a patient agent reaches a road it will continue to ‘Walk’ on a road cell until it encounters a car agent which gives the person a lift to a destination point. The model allows the comparison of purely raster based travel time and that with a variable network traffic flow.

### Response from users in a developing country

In order to understand how these modelling tools have been used to inform policy and planning, the authors conducted two sets of interviews. The first interview and focus group discussion took place in Bali (4–5 November 2016] while the second interviews took place in West Timor (TTS District) [23 January 2017]. The findings from these interviews are presented in section "Using SAGA-GIS and netlogo to inform policy".

## Results

### Accessing the tools

The GIS tool kit is available within the current releases (2.5+) of SAGA-GIS software available at: https://sourceforge.net/projects/saga-gis/. A tutorial video and manual for using the SAGA tool kit are available here; https://rohanfisher.wordpress.com/travel-time-modelling-saga/. The geo-simulation tools can be downloaded and a demonstration video is accessible here: https://rohanfisher.wordpress.com/modelling-access-to-services/. These tools are designed so they can be modified to suit differing contexts and applications and are thus open access.

### SAGA travel time tools

Conducting travel time analysis with the SAGA chain tool substantially simplifies the process of producing the land cover speed and the final travel time grids. The fact that the data were freely available and easy to obtain and the software’s open-access facilitated the decentralised application of this form of analysis. Although originally developed independently, as an output of this research, the code for these tools has been optimised and incorporated into SAGA GIS as part of its standard tool set since version 2.5. SAGA with the travel time chain tool can be downloaded through the projects source forge repository [41]. The tool can be found under the grid analysis travel time menu.

### Geo-simulation model 1 (distance transform implementation)

**Fig. 5:**
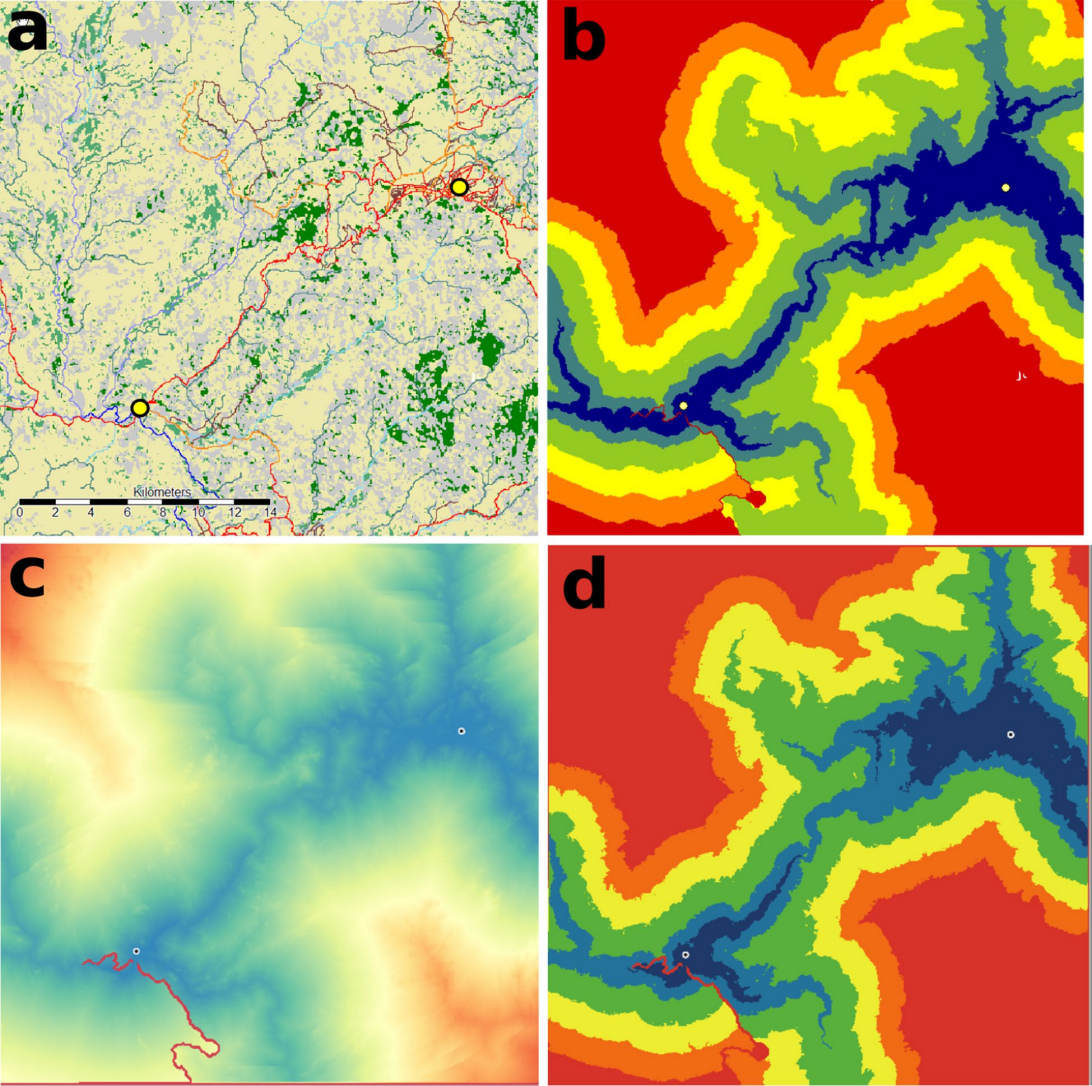
A comparison of outputs from the GIS and ABM travel time models: **a** the base land cover grid data and destination points, **b** travel time zones modelled in SAGA GIS (0–15 min *dark blue*, 15–30 mins *mid blue*, 30–60 min *green*, 60–90 min *yellow*, 90–120 *orange*, >120 *red*), **c** travel time continuous grid modelled in Netlogo, **d** travel time zones model Netlogo (Zone colours same as in **b**)

A comparison, using the same input data, of the output resulting from travel time analysis conducted using the SAGA-GIS tool to that produced by the geo-simulation can be seen in Fig. 5. It is clear from this that, whilst there is some difference between the shape and zone extents, the differences are minor and probably due to differences in the travel time calculation algorithms employed. This assessment provides confidence that the geo-simulation is producing valid results based on the input data.

**Fig. 6:**
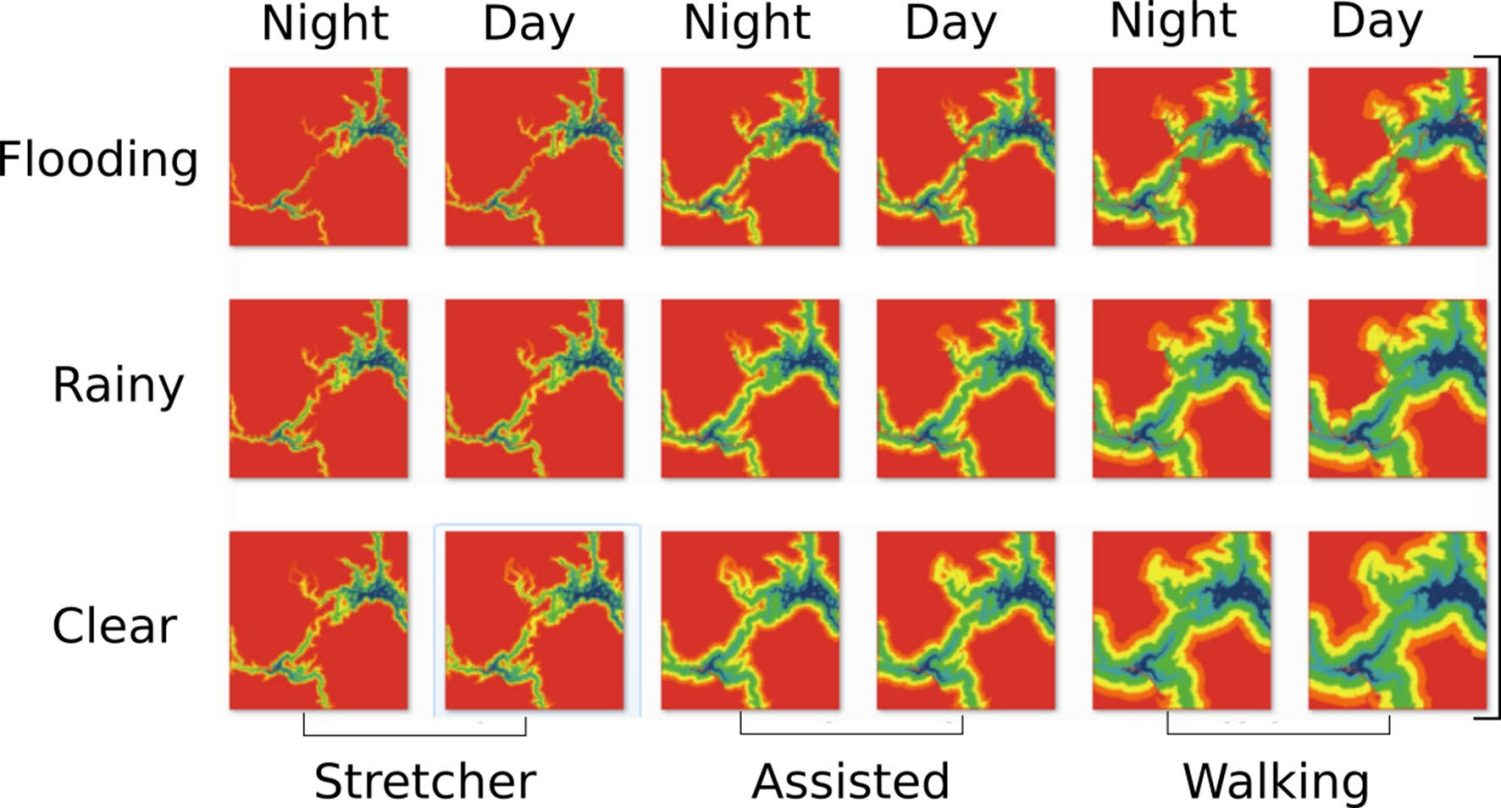
Netlogo ‘Behavior Space’ output. Eighteen models of travel time to two destination points. Travel zones are shown with the worst travel conditions at the *top left* to the best at the* bottom right*

A key advantage of the travel time implementation with a simulation platform is the ability to run the model using multiple variable settings. Most ABM platforms allow the automation of multiple model runs; within Netlogo this is known as the ‘Behavior Space’. Using this function with the model described here, it is possible, for example, to automate 18 model runs based on three walking travel speeds, two times of day and three weather conditions producing graphic outputs for each version. This is shown as a matrix of possible travel scenarios in Fig. 6.

### Geo-simulation model 2 (raster-network)

**Fig. 7:**
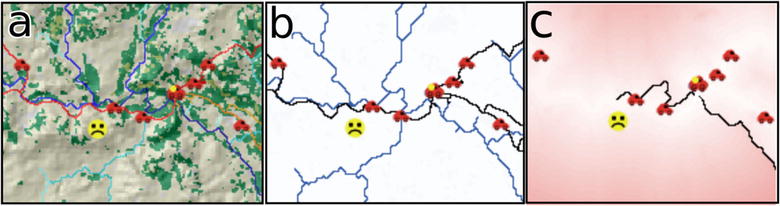
Screen shot from the Raster-Network Netlogo travel Model with **a** shaded relief land cover underlay image, **b** road and river network underlay and **c** shaded travel time grid. The ‘sad face’ icon represents an ill person travelling to a health service

This Netlogo model combines raster and network travel modes incorporating the GIS cost-distance output to create an interactive ‘gaming’ environment around travel time. The simulation used a ‘sad face’ icon to represent the ill patient travelling cross country towards a road and cars moving along the road network. Three visualisation images can be selected showing a shaded relief image and the raster grid and network features governing the model (Fig. 7). In addition, the model interface allows traffic intensity to be altered to simulate different times of day. Also shown is the distance travelled, time travel and average speed. The capacity to change the views and traffic parameters is designed to help build improved understanding of how the underlying modelling operates.

## Using SAGA-GIS and Netlogo to inform policy

Whilst other tool sets have been developed to assist travel time analysis [28], this is the first to be based around free open source software. The SAGA-GIS and Netlogo travel time tools can make the process of access analysis simpler than that available in proprietary software. The relative simplicity and functionality of these tools has the potential to support the decentralisation of travel time assessments. Both tools have been recently introduced to health planners in Eastern Indonesia in the South Central Timor (TTS District) of East Nusa Tenggara province which is well known as an underdeveloped and a disaster prone region. In this district two health officials and one local disaster management official were provided the Netlogo and Saga SDSS tools to inform policy options related to investment in both transport infrastructure and health facilities.

**Fig. 8:**
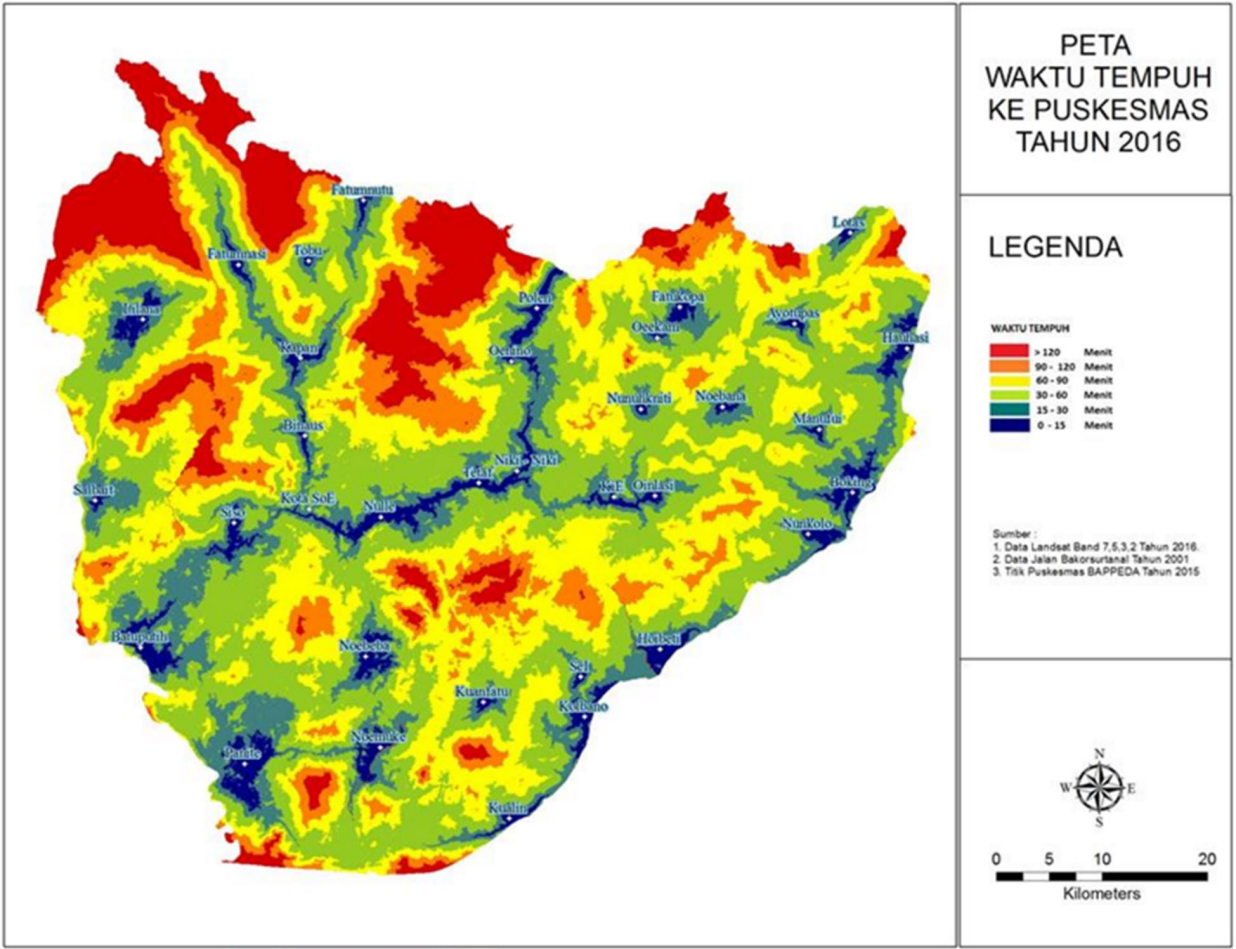
Travel time to clinics as produced by the subdistrict local government of Timor Tenggah Selatan using the SAGA-GIS travel time tools to support planning for the development of new health infrastructure. The* figure* shows travel time as zone increments from <15 min in *dark blue* to greater than 2 h in *red*. *Source* [46]

Those introduced to the tools mentioned ease of use as one of the key enabling variables supporting their uptake [44, 45]. In TTS district, resulting travel time visualisations, as shown in Fig. 8, have been used to inform local and national government investment plans for health infrastructure. These tools were used to support a clear evidence-based assessment of the geographic vulnerability of communities in the region. The vulnerability visualisation enabled local health to advocate to the local development planning unit, senior local officials and national health officials to build a new hospital and health facilities in a remote sub-district [44–46].

The toolkits have been important for guiding the local and national health planners to think about the strategic placement of new infrastructure, particularly with regard to communities vulnerable to flooding. In TTS district, monsoonal flooding in December and January is often followed by access disruption due to damaged roads and roads being cut by landslide debris. Therefore, according to the local officials, the travel time tools helped them identify not only alternative routes to health facilities but also guide local disaster management offices to identify vulnerable areas that can be potentially damaged by both floods and landslides.

Whilst initially developed for health planning, the tools and some training was also provided to one local emergency planner who subsequently used the SAGA GIS toolkit to develop local risk and vulnerability map for five villages. However this disaster risk modelling did not incorporate the net logo tools due to different reporting requirements between local health and emergency departments [45].

## Discussion

While the promise of the use of technology and SDSS for increasing both efficiency and effectiveness of government planning in the developing world is well recognised, the uptake of the SDSS have been often challenged by the quality of human resources [43] and the usability and practicality of SDSS [45, 47]. Furthermore the planning information provided by these tools is sometimes not fully understood by policy makers [44, 47]. The approaches described in this paper have been designed to overcome some of the limitations common in analysis of health care access. The tools described reduce some of the barriers to the more wide-spread use of GIS analysis of service access, allow complex spatial and temporal variations in service availability and travel speeds to be explored, facilitate a more complex assessment of multi-modal travel, allow the exploration of individual cases and support the integration of local knowledge.

Another advantage of the SAGA travel time tool is the integration of a number of pre-existing tools that facilitate the development of input grids. For example SAGA provides a comprehensive set of image classification tools for producing the vegetation grid layer from free Landsat data. SAGA also provides simple tools for incorporating elevation derived metrics, such as the Strahler stream orders, into the process. The travel time implementation in SAGA is robust, fast and transparent. Large datasets can be analysed quickly and the underlying inputs and variables can be easily queried and altered. The analysis outputs can also be exported to other GIS software for further work. The toolchains used to automate the processes are also reasonably simple to comprehend and alter. This is important as different regions will have different travel condition parameters not currently incorporated into the tool chains. For example in very rugged places it would be easy to add another step within the travel time tool chain to take into account slope for modifying travel speeds.

Whilst clearly a useful tool the GIS, implementation is only appropriate when set up and mediated by someone with familiarity with GIS and specifically SAGA GIS functions. The NetLogo models were therefore developed to provide a user friendly interactive environment from which to work with the GIS derived raster data.

### Geo-simulation model 1 (distance transform implementation)

To the author’s knowledge this is the first implementation of a distance transform algorithm for health access modelling within an ABM platform. The key benefit of the ABM implementation is the way it facilitates rapid and flexible modelling of multiple travel time scenarios through a simple user interface. Using the range of pre-set parameter settings along with the road, barrier and services feature drawing tools allows for an unlimited number of possible scenarios to be explored. The geo-simulation model makes it a relatively easy process to teach anyone how to parameterise and ‘play’ with access scenario modelling. The speed at which parameters can be altered and new scenarios explored has the potential for the model to fit into infrastructure planning discussions. Furthermore the ability to automate the production of multiple scenarios, as shown in Fig. 7, enables multiple dimensions of travel to be visualised. These can be produced in both image or GIS data formats for presentation or analysis independent of the ABM platform.

Despite its flexibility, the model described here has limitations related to the allowable size of the input grids and the processing speed. The NetLogo platform is Java based running in RAM memory. This can result in larger grid models, i.e. greater than 1000 × 1000 cells, being unable to load into NetLogo without editing Java parameters. The chamfer cost analysis used in this ABM takes double the time to conduct a grid analysis than when the same data is analysed in SAGA GIS. However, neither of these issues is considered a significant barrier to the useful application of the model. The grid size limitation still allows large areas to be modelled and processed at reasonable speeds. The model, for example, presented in this paper uses a 30 × 30 km grid with a 50 m cell size but conducts a travel time analysis in ~8 s. Grid size and processing speed could be increased with further code optimisation and the use of implementation platforms such as python.

### Geo-simulation model 2 (raster-network)

This model allows local knowledge to be used to place people into the landscape thus simulating individual cases. This can be useful particularly where population distribution data is not available. This model could easily be extended to allow the comparison of multiple travel conditions i.e. two agents—one walking at night, the other being stretchered during the day—could leave the same point at the same time and their progress monitored. In addition more complex network rules could be applied supporting different route paths and different travel speeds for a range of vehicle types. Ambulance travel requiring a journey from the destination point to the agent and back to the destination point could also be modelled. Whilst currently the model uses a grid layer to mimic network movement, it would be possible to incorporate a real network layer into the model, more accurately representing network function.

### Further work

The SAGA and geo-simulation travel time modelling tools could be further extended to include more service access factors. For example it is known that cell phones are critically important in many situations for accessing care [13], being used to contact health service professionals and to gain help when needing to travel to a health facility. Therefore a lack of mobile phone signal could add another layer to remoteness analysis. Modelling potential signal coverage could easily be added into the SAGA tool chain by incorporating available terrain tools. Similarly signal coverage could be incorporated into the geo-simulation through the incorporation of elevation data and code that models signal travel.

## Conclusion

Travel and service access is complex, not reducible to a few static modelled outputs [9, 10]. The approaches described in this paper use a unique set of tools to explore this complexity, promote discussion and build understanding in order to produce better planning outcomes.

The potential benefits of health GIS are often constrained by access to basic health information and a lack of skills among public health personnel for interpreting spatial data [8, 42]. In addition, current spatial models of health care access simplify the multifaceted and complex nature of real world travel. The accessible, flexible, interactive and responsive nature of the applications described here, through facilitating interactive scenario gaming and the discussions they provoke, have the potential to allow complex environmental social and political considerations to be combined and visualised. The further development and integration of this approach has the potential to make the governance and planning of service delivery more open, transparent and effective. Whilst some of the health service access issues incorporated into these models may not be relevant in all contexts, this paper describes an open and easily adaptable approach to developing interactive spatial decision support systems for health planning.

In emergency management studies, lead time is often defined as the time difference between the initiation and completion of a natural hazard event. Having appropriate technology locally available can lower the time needed to access services and reduce loss of life and assets. In the context of disaster early warning systems, local emergency responders need to have better prediction of both lead time and travel. The former will lead to better preparedness and the later will lead to better response planning [43]. Alternatively these applications could be used for modelling access and remoteness for a broad range of planning and governance tasks such as assessing remoteness from markets and schools or for modelling travel time. Indeed there is considerable scope for further exploration of the role of integrating GIS and interactive geo-simulations to support a range of planning, education and advocacy roles. In particular the opportunities for interactive geo-simulation applications in supporting evidenced-based service provision and participatory planning in both the developed and developing world are yet to be fully explored.
